# Sex-specific differences in DNA double-strand break repair of cycling human lymphocytes during aging

**DOI:** 10.18632/aging.203519

**Published:** 2021-09-10

**Authors:** Melanie Rall-Scharpf, Thomas W.P. Friedl, Shahar Biechonski, Michael Denkinger, Michael Milyavsky, Lisa Wiesmüller

**Affiliations:** 1 Department of Obstetrics and Gynecology, Ulm University, Ulm, Germany; 2 Department of Pathology, Sackler Faculty of Medicine, Tel Aviv University, Tel-Aviv, Israel; 3 Institute for Geriatric Research Unit, Agaplesion Bethesda Hospital, Ulm University, Ulm, Germany

**Keywords:** aging, sex, DNA double-strand break repair, BLM, end resection

## Abstract

The gender gap in life expectancy and cancer incidence suggests differences in the aging process between the sexes. Genomic instability has been recognized as a key factor in aging, but little is known about sex-specific differences. Therefore, we analyzed DNA double-strand break (DSB) repair in cycling human peripheral blood lymphocytes (PBL) from male and female donors of different age. Reporter-based DSB repair analyses revealed differential regulation of pathway usage in PBL from male and female donors with age: Non-homologous end joining (NHEJ) was inversely regulated in men and women; the activity of pathways requiring end processing and strand annealing steps such as microhomology-mediated end joining (MMEJ) declined with age in women but not in men. Screening candidate proteins identified the NHEJ protein KU70 as well as the end resection regulatory factors ATM and BLM showing reduced expression during aging in women. Consistently, the regulatory factor BLM contributed to the MMEJ proficiency in young but not in old women as demonstrated by knockdown analysis. In conclusion, we show that DSB repair is subject to changes upon aging and age-related changes in DSB repair are distinct in men and women.

## INTRODUCTION

Aging is an inherently complex process, our understanding of which is limited. Two phenotypic aspects of this complexity are the gender gap in life expectancy of on average 4 years (WHO, 2019), as well as varying incidences of age-associated diseases, such as cardiovascular, neurodegenerative, autoimmune and malignant diseases [1], both suggesting differences in the aging process between the sexes.

Genomic instability has been defined as one of the hallmarks of aging [2], as it is accompanied by an accumulation of genetic alterations including point mutations, large chromosomal rearrangements and attrition of telomeres [3]. Moreover, a higher load of DNA damage can be observed in different primitive and mature cell types of aged organisms including humans [3–5].

In order to deal with DNA damage, cells have acquired various mechanisms, covering diverse DNA repair pathways, cell cycle arrest, apoptosis and underlying control by signal transduction pathways, collectively known as the DNA damage response (DDR) [6]. DSB, representing the greatest danger to genome integrity, can be induced by exogenous sources such as ionizing radiation (IR) or as a consequence of endogenous replication stress [7]. The most prevalent pathway for the repair of DSB, classical NHEJ, is active throughout all cell cycle phases [8]. However, during S- and G2-phase MMEJ, single-strand annealing (SSA) and homologous recombination (HR), which require DNA end resection to generate 3’ single-stranded DNA overhangs for the search of homologies, are favored [9]. Limited, initial end resection by the MRE11-RAD50-NBS1-CtIP complex is sufficient for MMEJ [9]. Long stretches of ssDNA produced by extended DNA end resection by EXO1 or DNA2-BLM/WRN, are required for SSA or HR [8]. While HR allows error-free repair by copying sequence information from the homologous sister chromatid, SSA and MMEJ involve annealing of repeats within the two resected DNA strands resulting in loss of the sequence in between.

A decline in the overall capacity to repair DNA damage with age has been reported in different studies [4, 10, 11]. Importantly, the existence of premature aging syndromes caused by DNA repair gene defects supported the theory that the accumulation of DNA lesions due to DNA repair defects accelerates aging [12]. However, only few studies addressed specific pathway usage in human cells [13–15]. To our knowledge no data exists on differences in DSB repair pathway activities in men versus women during aging. In fact, differences between the sexes, have neither been adequately addressed in DDR mechanisms nor in the aging process so far.

Thus, to investigate whether capacity and/or fidelity of DNA repair change during aging and whether such changes are distinct in men and women, we analyzed DSB repair in primary peripheral blood lymphocytes (PBL) from male and female donors of a young and an old age group. Monitoring specific DSB repair activities revealed diametrically opposed changes in the NHEJ activity with age in PBL from men and women. Moreover, women showed a decline in end resection-mediated DSB repair pathways during aging. Protein analysis suggests a decline in KU70 and BLM expression exclusively in female PBL during aging underlying sex-specific changes in age-associated genome stability.

## RESULTS

### The activities of specific DSB repair pathways change in PBL with age in a sex-dependent manner

DSB repair pathway usage was analyzed in *ex vivo* cultured, cycling lymphocytes isolated from peripheral blood of young voluntary donors aged between >17 and 26 years and from elderly voluntary donors older than 60 years (Table 1). Given that replication stress, which has been identified as a potent driver of the aging process [5, 16], is a key endogenous source of DSB, and the full spectrum of repair pathways is used only in cycling cells, we induced proliferation in PBL, as it occurs e.g. in response to infections, and cultured them for 72h.

**Table 1:** Overview of study cohort.

**Group**		**n**	**Age**			**BMI**		
**Age group**	**Sex**		**Mean**	**Median**	**Range**	**Mean**	**Median**	**Range**
young	female	53	22.8	22.9	17.4-25.9	22.4	22.0	17.5-29.4
young	male	35	22.1	22.7	18.1-25.5	23.4	23.0	18.6-30.8
old	female	44	73.5	71.6	60.1-90.8	27.1	27.1	19.2-38.1
old	male	36	72.3	70.0	60.1-93.6	27.2	26.7	22.1-35.4

BMI, Body-Mass-Index; n.d., not defined; patients with malignant diseases during the last 3 years were excluded from the study.

**Figure 1:**
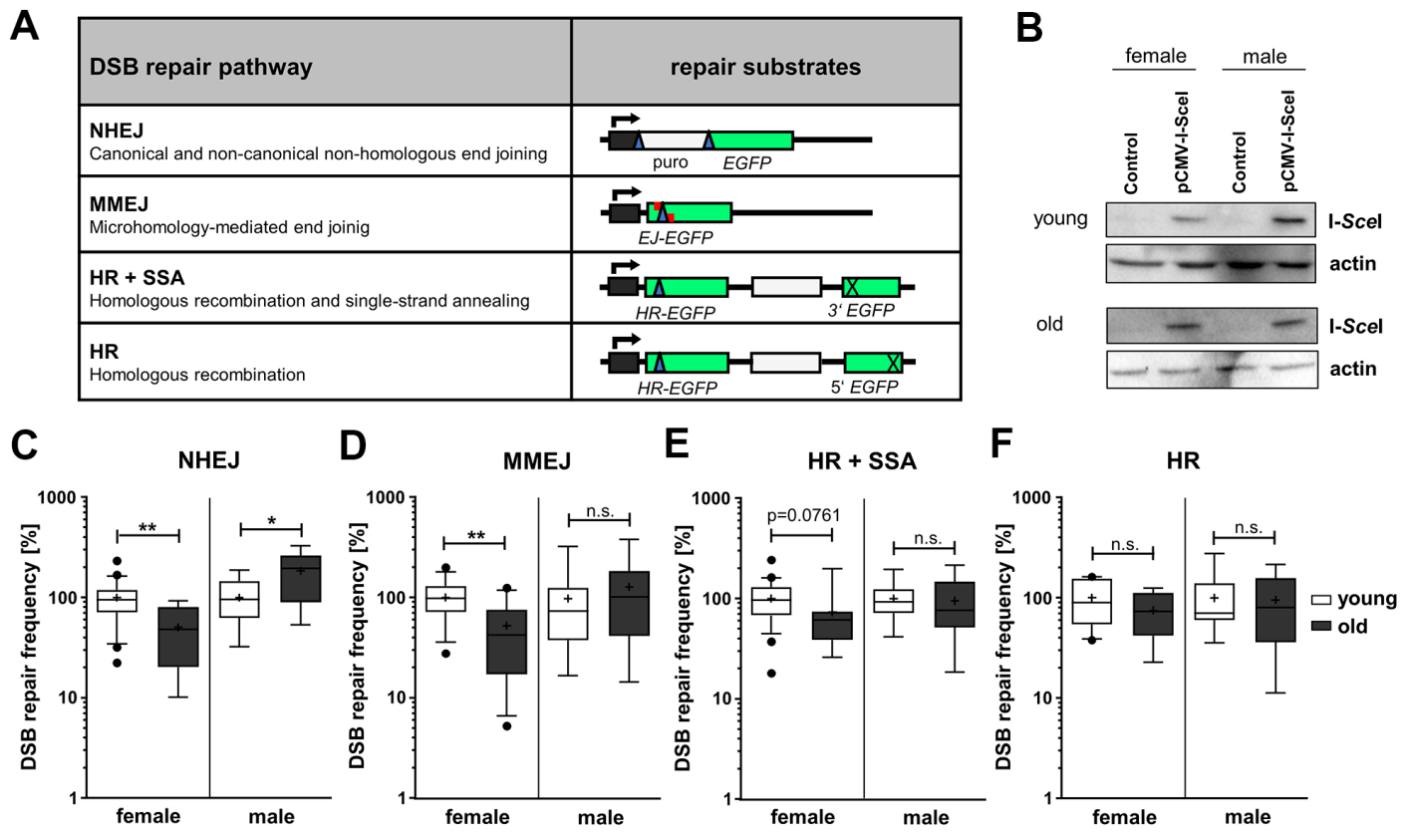
**DSB repair pathway activities in PBL from different age groups.** (**A**) DSB repair substrates. To detect NHEJ we used the reporter plasmid EJ5SceGFP, containing two tandem I-*Sce*I cutting sequences flanking a spacer separating the transcriptional promoter from the *EGFP* coding sequence. For MMEJ we employed substrate EJ-EGFP harboring a mutated *EGFP* gene, with an I-*Sce*I site flanked by 5 bp sequence repeats. Substrates HR-EGFP/3'EGFP and HR-EGFP/5'EGFP both contain *EGFP* lacking 4 bp at the position, where the I-*Sce*I site was inserted. For detection of homologous repair (HR+SSA) substrate HR-EGFP/3'EGFP additionally contains 3'*EGFP* mutated at the start codon, while HR substrate HR-EGFP/5'EGFP contains 3’ truncated 5’*EGFP*. I-*Sce*I site, blue triangle; cross, inactivating mutation/truncation; green bars, *EGFP* variants; white bars, spacer sequences; grey bar with kinked arrow, transcriptional promoter. (**B**) I-*Sce*I protein levels were analyzed by Western Blotting. Shown are representative blots of each age group and gender. (**C**–**F**) DSB repair activity measurements. DSB repair frequencies by NHEJ (**C**), MMEJ (**D**), HR+SSA (**E**) and HR (**F**) are shown in box plots with mean value (cross), median (line) and 95% Cl (whiskers). Data was generated from samples of 7-24 female and 7-15 male donors per age group and normalized to the mean of young donors for each sex group on the experimental day (Supplementary Table 1). The absolute mean DSB repair frequencies for young donors set to 100% were: (**C**) NHEJ, 8.00 x 10^-2^ (female) and 2.80x10^-2^ (male); (**D**) MMEJ, 0.12x10^-2^ (female) and 0.13x10^-2^ (male); (**E**) HR+SSA, 0.78x10^-2^ (female) and 0.49x10^-2^ (male); (**F**) HR, 0.19x10^-2^ (female) and 0.18x10^-2^ (male) (Supplementary Table 1).

To measure total NHEJ, MMEJ, homologous repair (HR+SSA) and HR-mediated repair of I-*Sce*I-induced DSB we used four differently designed *EGFP*-based reporter plasmids (Figure 1A) [17–19]. I-*Sce*I expression was verified in PBL samples for all sub-groups by Western Blotting (Figure 1B). This well-established DSB repair assay system engaging standard operating procedures with sample-specific correction of transfection efficiencies (Supplementary Figure 1) has previously been shown to robustly detect even subtle differences in DSB repair pathway usage such as in cells from heterozygous carriers of DSB repair gene mutations or in case-control studies [20–23]. Strikingly, DSB repair frequencies differed between age groups, but also between sexes with age (Figure 1C–1F). When comparing age groups regardless of the sex, we noticed a tendency towards reduced relative activities in the old age group compared to the young age group for MMEJ and homologous repair (HR+SSA), namely by 13% and 18%, respectively (Supplementary Figure 2A).

Stratification of the age groups into sex-dependent subgroups unveiled NHEJ activity changes with age that were diametrically opposed between the sexes during aging and therefore hidden before stratification. While in PBL from female donors NHEJ activity was significantly decreased by 50% in the old compared to the young age group, the NHEJ activity increased by 85% in the old age group in PBL from male donors (Figure 1C). This surprising finding was supported by the fact that also comparisons of the absolute DSB repair frequencies (Supplementary Figure 2B, see Supplementary Table 1) between the sexes revealed a pronounced difference between NHEJ in young women (8.03x10^-2^) compared to young men (3.00x10^-2^) as well as in old women (3.22 x10^-2^) compared to old men (6.64 x10^-2^). Thus, as young women featured a significantly higher NHEJ frequency than young men, the age-related decline in women results in comparable NHEJ frequencies in old women and young men. Moreover, the sex-specific stratification revealed an age-related decline in MMEJ by 48% (Figure 1D) and indicated a trend (p value < 0.1, see Supplementary Table 1) towards a decrease in HR+SSA by 27% (Figure 1E) in PBL from women. Comparisons of the absolute DSB repair frequencies suggest even a significant decrease of HR+SSA (Supplementary Figure 2B). No changes in the activities of these end resection-dependent pathways were observed in men, and HR seemed not to be affected by age in both sexes.

**Figure 2:**
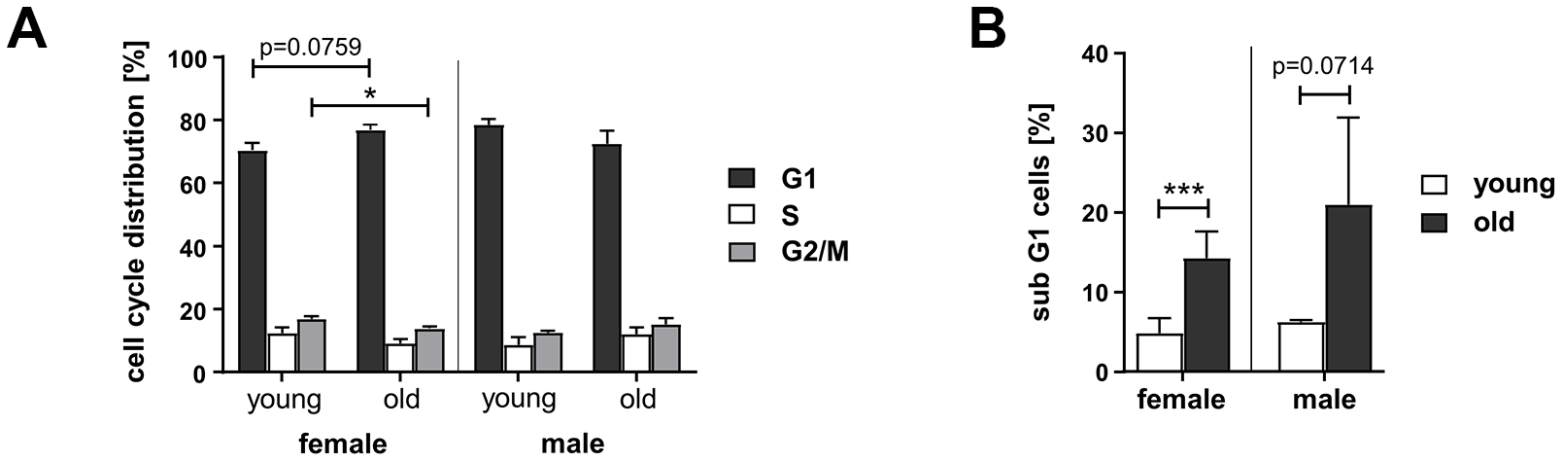
**Cell cycle distribution and cell death in PBL from different age groups.** Cultivated PBL were fixed and DNA content analyzed by propidium iodide staining and flow cytometry. Percentage of live cells in G1-, S-, and G2-phase (**A**) and proportion of dead cells, determined by subG1-DNA content (**B**); female: n=11 (young), n=8 (old); male: n=2 (young), n=6 (old) (Supplementary Table 1).

Since homologous DSB repair pathway choice is cell cycle regulated [24], we measured cell cycle distribution and cell death. In PBL from old women the proportion of cells in G2/M-phase was reduced by 18% compared to young women (Figure 2A and Supplementary Figure 3A), while in PBL from old men no changes in cell cycle distribution were apparent. The proportion of dead cells, detected as subG1 fraction, was increased in PBL from the old donor group up to 14% in women and, though not significantly, up to 21% in men (Figure 2B and Supplementary Figure 3B). Overall, we detected minor fractions of dead cells and changes in cell cycle distribution that are unlikely to fully account for the age-related and gender-specific changes in DSB repair pathway usage.

In conclusion, our analysis revealed sex-dependent changes in DSB repair pathway usage with age: a diametrically opposed change in the NHEJ activity with age in men and women and a female-specific decline in MMEJ and to a lesser extent homologous repair, i.e. pathways which require DNA end resection.

### Kinetics of IR-induced γH2AX and 53BP1 foci numbers suggest efficient DSB removal in PBL from old donors of both sexes

**Figure 3:**
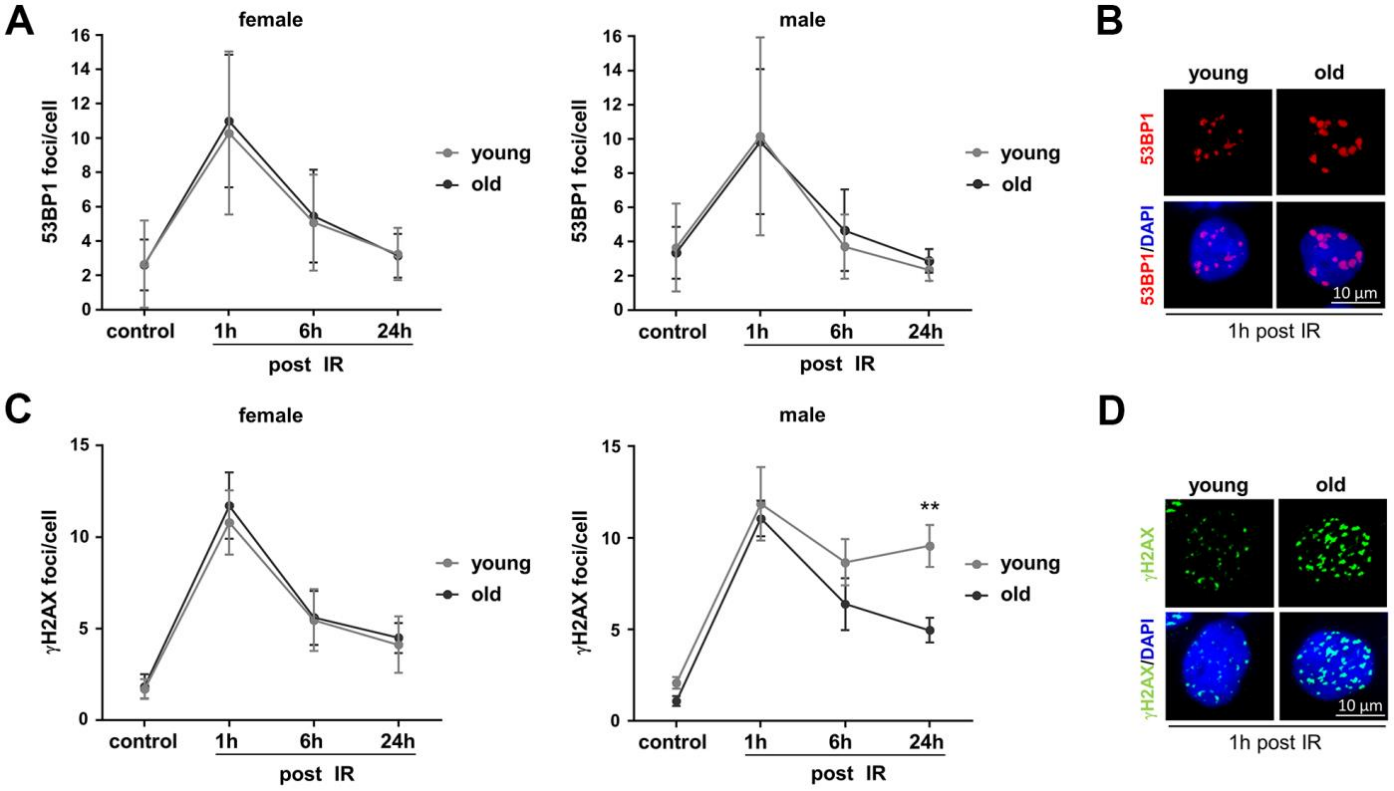
**Monitoring basal and IR-induced DNA damage in PBL from different age groups.** Irradiated PBL were re-cultivated and fixed at indicated time points. 53BP1 and γH2AX foci were detected by immunofluorescence microscopy and 50-200 nuclei per donor scored. (**A**) 53BP1 foci. Dots, mean values; bars, SEM; female: n=14 (young), n=11 (old); male: n=5 (young), n=7 (old) (Supplementary Table 1). (**B**) Exemplary immunofluorescence images of nuclei with IR-induced 53BP1 foci. (**C**) γH2AX foci. Dots, mean values; bars, SEM; female: n=8 (young), n=8 (old); male: n=8 (young), n=8 (old) (Supplementary Table 1). (**D**) Exemplary immunofluorescence images of nuclei with IR-induced γH2AX foci.

Besides analyzing specific DSB repair activities, we investigated overall DNA damage removal. To this end we monitored IR-induced 53BP1 and γH2AX foci in PBL engaging quantitative immunofluorescence analysis (Figure 3). The kinetics of foci assembly and disappearance in PBL from all groups show that 53BP1 as well as γH2AX foci numbers significantly increased 1h post IR from control levels and then declined indicating repair of DNA damage. While 53BP1 foci numbers (Figure 3A) returned to the level of unirradiated control cells within 24h post IR, basal levels of γH2AX foci (Figure 3C) could generally not be reached within this time frame, indicating residual damage or repair intermediates that are not marked by 53BP1.

Comparison of the age groups revealed on average comparable basal and IR-induced 53BP1 foci numbers per nucleus in PBL from young and old donors of both sexes (Figure 3A, 3B). Likewise, no significant differences of basal and IR-induced γH2AX foci could be observed in PBL from old compared to young donors (Figure 3C, 3D), except that young male donors showed elevated γH2AX foci numbers 24h post IR. Since IR-induced DSB are thought to be mostly repaired by NHEJ [25], higher γH2AX foci numbers in young men might at least in part be explained by their lower absolute NHEJ activity compared to young women and old men (Supplementary Figure 2B). Yet, the decline in NHEJ activity in old women to the level of young men (Supplementary Figure 2B) did not result in a corresponding increase in γH2AX foci numbers (Figure 3C).

Of note, 53BP1 is known to protect DSB from end resection and mediates NHEJ, while phosphorylation of γH2AX is also present at resected DSB [26] and other DNA lesions including sites of replication stress [27]. Colocalization of both markers, determined as percentage of γH2AX foci overlapping with 53BP1 foci, was higher in PBL from young male versus female donors 6h and 24h post IR (Supplementary Figure 4). Thus, colocalizing foci largely followed the kinetics of γH2AX foci (Figure 3C) indicating similar differences of unrepaired DSB and overall damage between these two groups. A higher percentage of γH2AX foci colocalizing with 53BP1 was as well seen in PBL from old men compared to old women 24h post IR (Supplementary Figure 4) despite the concomitant decline of γH2AX foci in PBL from both old donor groups (Figure 3C). Since NHEJ was higher in PBL from old men versus old women (Supplementary Figure 2B, see Supplementary Table 1), the high percentage of colocalizing 53BP1 foci in PBL from old men may reflect preferred use of this DSB repair pathway.

Collectively, 53BP1 and γH2AX foci analysis does not suggest that the overall capacity to repair DSB post IR is compromised in PBL with age, however, supports the concept of age-related changes in DSB repair pathway usage in men.

### Decline in KU70, BLM and ATM expression with age in PBL from women

**Figure 4:**
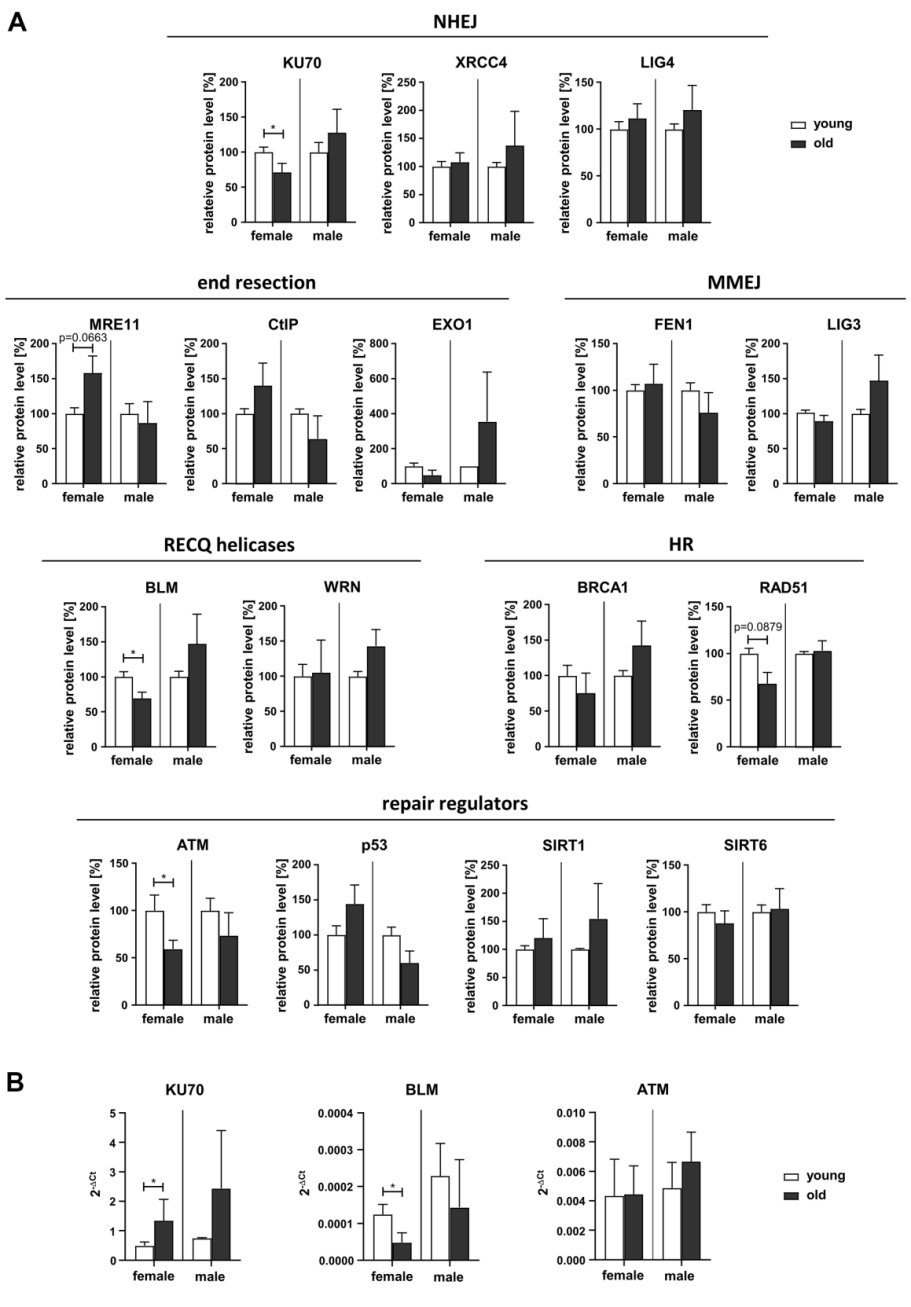
**Expression of DDR proteins.** (**A**) Protein levels of DDR factors were determined by Western Blotting (representative blots are shown in Supplementary Figure 5). Protein band intensities were quantified and normalized to loading controls. Normalized values for PBL derived from young female or male donors respectively were set to 100% for each blot. Columns, means of relative protein levels; bars, SEM from 1-11 donors per age group (Supplementary Table 1). (**B**) mRNA levels of KU70, BLM and ATM determined by RT-qPCR. Columns, mean 2^-ΔCt^ values; bars, SD; female: n=3 (young), n=5 (old); male: n=3 (young), n=4 (old) (Supplementary Table 1).

In order to understand the underlying molecular causes for the observed changes in NHEJ and end resection-mediated DSB repair pathways we analyzed the relative protein levels of candidate factors [28] by Western Blotting (Figure 4A and Supplementary Figure 5). In contrast to previously published results on fibroblasts [13], we found no changes in the protein levels of the classical NHEJ or MMEJ factors LIG4, XRCC4, LIG3 and FEN1 in PBL with age. However, KU70 levels were reduced in PBL from old female but not male donors, compared to PBL from young donors. Since the KU70/KU80 heterodimer is a crucial mediator of classical NHEJ, reduced KU70 levels can clearly explain the NHEJ decrease in old women (Figure 1C). Quantification of the proteins mediating initial end resection (MRE11, CtIP) indicated a close to significant increase of MRE11 in PBL from female donors with age, even though end resection activity decreased with age. However, among the analyzed factors promoting more extended end resection (EXO1, BLM, WRN) reduced BLM levels were found in PBL from old versus young women. ATM levels also declined in old compared to young women, possibly contributing to the trend towards a decrease in homologous repair (Figure 1E). Other regulators of DNA repair (p53, SIRT1, SIRT6) or core HR proteins (BRCA1, RAD51) did not show significant changes with age, though a trend to reduced RAD51 levels was noticed in old versus young women.

Altogether, Western Blot analysis revealed reduced expression levels of KU70, ATM and BLM in old female donors targeting NHEJ and repair of more complex DSB, respectively.

In addition, we assessed mRNA levels of factors showing significant changes in protein level (KU70, BLM and ATM) by RT-qPCR (Figure 4B). While ATM mRNA levels were comparable regardless of sex or age, KU70 mRNA levels were increased in old compared to young females suggesting these two proteins are downregulated by posttranscriptional mechanisms. In contrast, BLM mRNA just like BLM protein levels showed a decline in old versus young females.

### BLM depletion decreases MMEJ-mediated DSB repair in PBL from young female donors

**Figure 5:**
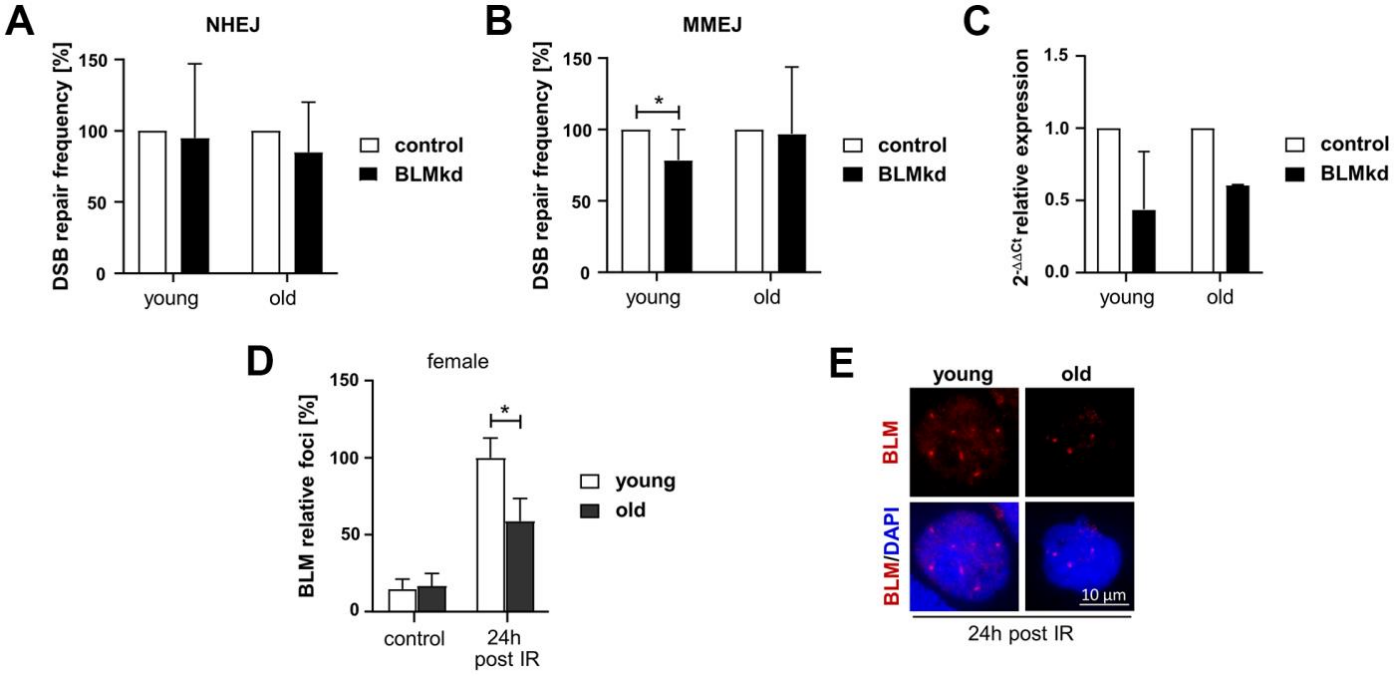
**Analysis of BLM-dependent DSB repair and BLM foci formation in PBL from female donors.** (**A**–**C**) DSB repair activity measurements. Cultivated PBL were nucleofected with a DNA mixture containing pCMV-I-SceI, repair substrate EJ5SceGFP (NHEJ) (**A**) or EJ-EGFP (MMEJ) (**B**), pBS or wild-type EGFP expression plasmid and knockdown (kd) plasmids silencing BLM or empty vector controls. Mean values for samples nucleofected with control plasmid were set to 100% for each donor. Columns, mean values; bars, SD; n=4-9 donors; *, p<0.05; Wilcoxon matched-pairs signed rank test (Supplementary Table 1) (**C**) quantitative PCR analysis of BLM expression to validate knockdown efficiency. Columns, mean relative expression; bars, SD; female: n=4 (young), n=2 (old). (**D**, **E**) BLM foci formation. BLM was immunocytochemically detected 24h post IR. Foci numbers of 50-200 nuclei per donor were scored. (**D**) Columns, mean values; bars, SEM; female: n=13 (young), n=11 (old) (Supplementary Table 1). (**E**) Representative immunofluorescence images of nuclei with IR-induced BLM foci.

As the helicase BLM, interacting with the MRE11-RAD50-NBS1 complex downstream of ATM, is required for the recruitment of NHEJ factors [29] and coordinates DNA end processing [30], its reduced expression in PBL from old women makes BLM a top candidate to impact on both NHEJ and MMEJ, the pathways we saw the greatest differences between the age groups. In order to link reduced BLM expression to the age-related changes in DSB repair, we transiently introduced a previously established BLM-specific shRNA [31] in PBL from female donors and measured NHEJ and MMEJ-mediated repair of I-*Sce*I-induced DSB (Figure 5A–5C). BLM knockdown reduced expression by about 50% in PBL from young and old donors as verified by RT-qPCR (Figure 5C).

Interestingly, reduction of BLM expression seemed to have no effect on NHEJ activity in PBL, neither from young, nor from old female donors (Figure 5A). In contrast, BLM knockdown reduced the MMEJ activity in PBL from young women, while it did not affect MMEJ in PBL from elderly women (Figure 5B). Thus, reduction of BLM expression in PBL from young women mimics the phenotype observed in PBL from old women, suggesting that declining BLM levels are causally linked to reduced MMEJ activity in elderly women. To exclude the possibility that reduction of BLM level affects cell cycle progression, we analyzed the cell cycle distribution in PBL samples from young and old women after BLM knockdown (Supplementary Figure 6).

Since BLM seemed to be the most promising candidate to explain the changes in DSB repair in women, we further investigated the assembly of BLM in nuclear foci in response to IR by quantitative immunofluorescence analysis of these cells (Figure 5D, 5E). BLM foci numbers markedly increased 24h post IR, likely indicating BLM recruited to DSB arising during replication [32]. Yet, PBL from old women showed significantly reduced BLM foci numbers confirming impaired BLM function with age.

## DISCUSSION

Until recently, sex differences have largely been neglected in the DDR-related aging research. First pieces of evidence for sex-specific differences in DNA repair have been provided such as a greater mutation load in men than in women [33] and sex-differences in oncogenic mutational processes [34]. Here we identify differences between the sexes in age-related changes in DSB repair in cycling human PBL.

### Differential regulation of NHEJ activity during aging in PBL from men and women

DSB repair by NHEJ showed the most pronounced sex differences, as it was inversely regulated during aging in men and women. While NHEJ declined in PBL from women of the old age group compared to the young age group, it strongly increased with age in PBL from men. Since IR-induced DSB are mostly repaired by NHEJ, these findings are compatible with an earlier study reporting a more pronounced age-related decline in re-joining of DSB, induced by 30 Gy of X-ray, in PBL from women than from men [10]. Conversely, Garm et al*.* [11] found no sex differences in age-associated changes in the capacity to repair 6 Gy-induced DSB in human peripheral blood mononuclear cells. Yet, both studies did not analyze specific DSB repair pathways.

Li et al*.* as well as Anglada et al*.* [13, 15] described a decline in NHEJ using an assay comparable to our system, however, in cells from female donors only. While these female-specific findings are similar to our results, different molecular causes seem to mediate changes in NHEJ activity in eyelid fibroblasts [13] and PBL (Figure 1). We could not detect a reduction in the protein levels of LIG3, LIG4 or XRCC4, but found reduced levels of another key NHEJ factor, KU70, in old female donors. A decline of KU70 protein levels with age has previously been recognized, though in a group of donors with unknown sex composition [35]. Moreover, depletion of KU70, or other crucial NHEJ factors like KU80 and DNA-PKcs in mice, known to cause immunodeficiency, also leads to an earlier onset of aging-related pathologies and a significantly shorter lifespan compared to wild-type animals [36, 37]. In human mammary epithelial cells (HMEC) deficient recruitment of 53BP1 in G1 phase was suggested as cause for the age-associated DSB repair defect, leading to binding of BRCA1 and excessive end resection without triggering HR [15]. We could however not observe reduced levels of 53BP1 foci in PBL from old donors.

Notably however, in independent studies we found a positive correlation between PARP activity and donor age in PBL from men [38], while no such correlation was found in women [22]. PARP1 is involved in classical and alternative end-joining [6], PARylation capacity has been associated with longevity [39] and found to be higher in men than women [40]. From this, declining KU70 levels might contribute to reduced NHEJ activity in PBL from old women, while increased PARP activity might promote NHEJ and maintain MMEJ activity in old men, collectively generating sex-specific differences in NHEJ in PBL from old donors.

### Decline of end resection-mediated repair pathways during aging in PBL from women

According to our DSB repair measurements HR+SSA shows a trend (p=0.0657, see Supplementary Table 1) to higher absolute frequencies in PBL from young women compared to young men, with a decrease in old women reaching a level comparable to both young and old men. This suggests young women repair DSB more frequently by HR+SSA-mediated repair than men and old women. Interestingly, in old women we observed an inverse relationship between the elevated level of MRE11, initiating end resection, and the diminished levels of ATM and BLM, controlling and promoting scheduled end resection [41, 42]. We propose that in old women reduced NHEJ permits access of initial end processing factors to DNA ends. However, improper end processing in the absence of ATM and BLM will lead to reduced reporter-based activities of the homology-mediated pathways MMEJ and HR+SSA. Li et al*.* and Anglada et al*.* [13, 15] described a decline in HR activity with age in cells from female donors due to hampered recruitment of the essential HR factor RAD51 [13]. Though not reaching statistical significance, we also calculated lower frequencies in PBL from old donors of both sexes and noticed a trend (p=0.0879) to a reduced RAD51 protein level in PBL from old female donors.

### Influence of sex hormones on DSB repair

The level of sex hormones, which is subject to age-related fluctuations, could contribute to sex-specific changes in DSB repair pathway activities. Among the sex hormones, particularly estrogens and metabolites are known to induce DNA damage and replication stress, ultimately resulting in stalled replication forks and DSB [43, 44]. As a consequence, young women may adapt to estrogen-induced DNA damage by activating NHEJ as well as homology-mediated DSB repair. Higher absolute DSB repair frequencies of NHEJ and HR+SSA in PBL from young women compared to young men may reflect such adaptation to persisting stress during the reproductive phase. Interestingly, the age-associated decline in estrogen levels might also impact on DSB repair by regulating BLM level, given that BLM gene expression is up-regulated by estrogen in a concentration-dependent manner [45].

In contrast to estrogen levels that drastically drop during menopause [46], testosterone levels continuously decrease in aging men [47], which might be one reason why no such decline in NHEJ and HR activity was detectable in men. Another reason may be related to the sex-differences in PARP activity, which have also been linked to sex hormones [40]. Of interest for this work, androgen receptor-mediated transcription was reported to promote various DNA repair mechanisms thereby preventing DNA damage accumulation [44, 48]. Since PARP1 is a key sensor of DNA damage [49], reduced androgen receptor signaling may explain increased PARylation with age in men [38], stimulating NHEJ as we observed here.

### Reduced BLM protein levels influencing DSB repair pathway choice might contribute to aging

Bloom’s syndrome, caused by null or missense mutations in the *BLM* gene, is characterized by genomic instability, increased occurrence of sister-chromatid exchanges, increased cancer susceptibility, insulin resistance and immunodeficiency [50], features that are also associated with normal aging. Moreover, BLM expression was found to be decreased in aged mouse hematopoietic stem cells [51]. Thus, it seems plausible that decreased BLM expression and impaired recruitment to damage sites, indicated by reduced assembly of BLM foci in PBL from old female donors, contributes to the aging process.

BLM functions in the DDR by multiple mechanisms, promoting as well as inhibiting different repair pathways depending on the cell cycle and repair phase [29]. Thereby BLM ensures timely and accurate repair to maintain genome integrity. Besides its role in extended end resection [52] BLM recruits multiple HR and NHEJ factors to DSB [29, 30]. Moreover, BLM stimulates DNA strand exchange activity of RAD51 [53], branch migration of recombination intermediates [54] and dissolves double Holliday junctions [30]. However, BLM can also inhibit HR by counteracting RAD51 loading [55] and disrupting D-loop structures after strand invasion [56]. In reporter-based assays for NHEJ, BLM was either found to exert a stimulatory [57], inhibitory [29, 58] or no effect [59, 60]. However, regardless of the NHEJ pathway analyzed, BLM was unequivocally found to suppress long-range (>200 bp) deletions that were explained by alternative end joining due to unscheduled end resection by CtIP and MRE11 [29, 41, 58, 59, 61]. Altogether, DSB repair dysfunction associated with BLM defects underlying the progeria Bloom's syndrome mark a prototypic link between DNA repair changes and aging [12].

Due to its multifaceted role it was unclear how the reduced level of BLM and impaired recruitment to damage sites seen in PBL from old women would affect different repair pathways. Starting from the observation that both NHEJ and MMEJ were significantly downregulated in old women (Figure 1), we focused on these pathways. Interestingly, our DSB repair measurements suggest BLM promotes MMEJ in PBL from young female donors, since knockdown led to a reduced frequency, mimicking the phenotype in old. In contrast no effect was observed with the NHEJ reporter that detects both classical as well as alternative NHEJ events.

In line with our observations are the findings by Langland et al*.* [61] on increased deleterious plasmid rejoining events in nuclear extracts from Bloom’s syndrome cells. These deletions were associated with reduced use of microhomologies, suggesting that BLM may be required for the precise alignment of the microhomology elements. Supporting this notion, Mendez-Dorantes and colleagues [62] demonstrated that BLM counteracts MMEJ with divergent sequences and promotes annealing of identical microhomologies, though in their repair substrates separated from the DSB by a long distance. Accordingly, we propose that PBL from old women, suffering from reduced BLM levels, disfavor repair processes involving such precise annealing events, as detectable by our MMEJ reporter, in favor of deleterious processes that may even destroy the reporter. As ATM directly interacts with, phosphorylates and recruits BLM to DSB [29, 63] and controls end resection common to MMEJ, SSA and HR [9, 29, 41, 42, 62], reduced ATM levels likely contribute to dysfunction in old women. While loss of controlled resection and precise annealing can explain DSB repair defects seen for MMEJ and for HR+SSA in PBL from old women, concomitant loss of the anti-recombinogenic effects of BLM may have blurred the picture seen for HR.

As the genomic instability induced by BLM loss in the germ line has a high impact on carcinogenesis, reduced somatic levels in PBL from healthy aged women very likely contribute to the increasing incidence of cancer with age. On the other hand, cancer -especially leukemia- more frequently affects men than women [64]. Yet also NHEJ, which is partially error-prone and increased in old men, might predispose to leukemia, as leukemia-associated chromosome translocations feature NHEJ-specific repair signatures [65].

### High overall DSB repair capacity at old age

While many studies describe increased basal and/or IR-induced γH2AX signal intensities with aging in mice and men [4, 16, 66–68], others observed a reduced γH2AX response in human beings [11]. Somewhat contradictory as well, decelerated [66] and accelerated [22] removal of γH2AX-labeled DSB with age were reported for HMEC from healthy female donors and breast cancer patients, respectively. In our work dual analysis of γH2AX and 53BP1 suggested that basal and IR-induced foci numbers are comparable between the age groups of both sexes and the overall capacity to repair IR-induced DSB seemed to be intact in PBL from old donors of both sexes. Surprisingly, PBL from young rather than old men retained residual γH2AX foci 24h post IR. Yet, supporting our findings, a decline of residual total γH2AX fluorescence signal per nucleus 24h post IR has also been observed in lymphocytes from 94 healthy volunteers with age, whereby old women had even lower residual levels than men [68]. Our aged probands were all healthy and not surprisingly their PBL dealt with IR-induced DSB still quite well. We believe that in contrast to severe repair defects caused by mutations in DNA repair genes leading to accelerated aging and increased cancer susceptibility [6], aging-related alterations of the DDR mechanisms might be rather small, however continuously leading to damage accrual driving the aging process.

In this context it is important to consider the general limitations of immunofluorescence-based approaches compared to reporter-based measurements. Detection of IR-induced foci only indirectly quantifies DSB repair by tracking the overall cellular response to DNA damage, while reporter-based assays monitor specific DSB repair pathways. Thus, it has been questioned whether γH2AX and 53BP1 foci disappearance completely coincides with DSB repair [69]. In addition, γH2AX foci do not only form at DSB but also at ssDNA occurring during replication, at telomeres, in nucleoli marking transcriptional silencing of rDNA genes and have been connected with senescence [16, 70–72]. Therefore, increased γH2AX foci might be associated with aging but not necessarily relate to unrepaired DSB und thus have to be interpreted with caution in this context.

### Repair of primary and secondary IR-induced DSB arising during replication

Given that primary IR-induced DSB are mostly repaired by NHEJ [25], PBL from young women as well as old men will rapidly repair DSB due to their highly active classical NHEJ activity, quickly reducing γH2AX as well as 53BP1 foci. In PBL from old women as well as young men with lower NHEJ activity, compensatory end resection will lead to a decline in 53BP1 foci. This can explain, why we could observe equivalent 53BP1 foci numbers in all groups irrespective of changes in pathway usage. Yet, clearance of γH2AX foci was delayed in young men, although old women showed comparable DSB repair activities. Thus, the question remains why young men show increased γH2AX foci numbers at later time points. Considering that IR does not only cause DSB but also other lesions including oxidative base damage, single strand breaks, fragmented sugar derivatives and loss of terminal base residues culminating in clustered damage or single stranded gaps [27], we speculate that the γH2AX foci observed at later time points in young men might represent secondary DSB that arise after fork stalling at these IR-induced lesions. Intriguingly, 53BP1 recruitment to DSB is suppressed in replicating chromatin [73], which could explain why only an accumulation of γH2AX but not 53BP1 foci could be observed. Therefore, unresolved γH2AX foci in young men might reflect DNA lesions and replication intermediates rather than clean DSB such as generated by I-SceI in reporter-based assays, which warrants in-depth analysis in future studies.

## CONCLUSIONS

Altogether, our findings suggest distinct sex-specific alterations in DSB repair pathway usage during aging that might contribute to the decline of genomic stability with age. While in women the activity of NHEJ declined upon aging, it became upregulated in men. Moreover, the activities of homology-mediated repair pathways decreased upon aging in women, while no such change was detectable in men. In PBL from old women, we found reduced expression of KU70 as well as ATM and BLM, which may contribute to the decrease in NHEJ and end resection-dependent pathways, respectively. However, changes in DDR during aging in men and women likely result from multiple subtle changes in the level, activity and recruitment of various repair factors, and are influenced by differential hormonal regulation and life-style. Collectively these changes contribute to the gender gap in life expectancy as well as in the incidences of age-associated diseases. Our work provides first pieces of knowledge for the development of individualized, gender- and age-specific therapeutic approaches, to protect genome stability during life enabling healthy aging.

## MATERIALS AND METHODS

### Collection, isolation and culture of primary PBL

Peripheral blood was collected with informed consent from young voluntary donors aged between >17 and 26 years and from elderly voluntary donors older than 60 years (Table 1). Part of the old donor cohort (14 donors) was recruited in the Agaplesion Bethesda Clinic Ulm within the framework of the ActiFE III study. Blood sample collections were approved by the local advisory board (approvals #105/2003; #157/10; # 393/16). Primary PBL cultures were generated as described [17], resuspended in PB-MAX™ Karyotyping Medium (Gibco) 2% Phytohemagglutinin (Gibco), at a cell density of 2×10^6^ cells/ml, and cultivated for 72h prior to all experiments.

### EGFP-based DSB repair assay

DSB repair pathway analysis was performed as described [17, 18]. PBL were transfected using the Amaxa B Cell Nucleofector Kit (Lonza). 10^6^ cells in 100 μl Amaxa B Cell nucleofection solution were nucleofected with 10 μg plasmid DNA per cuvette using program U-15. After nucleofection cells were immediately transferred into RPMI media (Gibco), containing 1% Penicillin/Streptomycin (Gibco), and 20% FBS (Biochrom). 2% Phytohemagglutinin (Gibco) was added only 4h post transfection. Plasmid mixes contained the I-*Sce*I expression plasmid pCMV-I-SceI, one of the DSB repair substrates EJ5SceGFP (NHEJ), EJ-EGFP (MMEJ), HR-EGFP/3’EGFP (HR + SSA) or HR-EGFP/5’EGFP (HR) (Figure 1A) and additionally pBlueScriptII plasmid (KS Stratagen) for DSB repair measurement, or wtEGFP expression plasmid for determination of transfection efficiency respectively (average transfection efficiencies: 11%). After re-cultivation for 24h PBL were harvested and analyzed via flow cytometry using a FACSCalibur™ (BD Biosciences). The fraction of green fluorescent cells within the whole live cell population (SSC/FSC gate) was measured by a diagonal gating method in the FL-1/FL-2 dot plot. Representative FACS plots are shown in Supplementary Figure 1. Each measurement in repair assays was individually normalized by the transfection efficiency corresponding to the specific sample to calculate the DSB repair frequency. Conditions chosen were previously established to ensure detection of EGFP signal changes in the linear range [18]. To silence BLM expression during DSB repair measurements, 2.5 μg of a pre-established shRNA expression plasmid (Origene) [31] were included into the nucleofection mixture.

### Immunofluorescence microscopy

To induce DNA damage PBL were exposed to a dose of 2 Gy γ-radiation. At the indicated times post irradiation cells were cytospinned on poly-L-Lysine (Sigma) covered glass slides and fixed in 3.7% formaldehyde (Th.Geyer) for 10 min. Fixed slides from all time points were collected after fixation. For immunofluorescence analysis of BLM and RPA foci cells were additionally pre-extracted in cold pre-extraction buffer (20 mM HEPES, pH 7.4; 50 mM NaCl; 1 mM EDTA; 3 mM MgCl_2_; 300 mM Sucrose; 0.5 % Triton X-100) for 1 min prior to fixation. For immunofluorescence staining slides were washed 3 times for 5 min in PBS and permeabilized with 0.5 % triton for 10 min. To avoid unspecific binding slides were blocked in 5% goat serum for 1h at room temperature, followed by immunostaining with primary antibodies, anti-53BP1 (rabbit, polyclonal, NB100-304, Novus Biologicals), anti-phospho-histone H2A.X (Ser139, mouse, monoclonal, JBW301, Millipore) or anti-BLM (rabbit, polyclonal, ab476, Abcam) diluted in 5% goat serum, at 37° C for 1h or at 4° C overnight. After another washing step (3 × 5 min in PBS) slides were incubated with secondary antibody Alexa Fluor555-anti-mouse, Alexa Fluor488-anti-mouse or Alexa Fluor555-anti-rabbit (Invitrogen) 1h at 37° C. Final washing (3 × 5 min in cold 0.1 % triton) was performed, before slides were mounted in Vectashield containing DAPI and sealed under cover slips. Nuclear immunofluorescence signals were imaged with a BZ-9000 microscope (Keyence) using a 100x objective. Automated identification and quantification of foci was carried out either using BZ-II Analyzer (Keyence) or Cell Profiler software [74].

### Cell cycle and cell death analysis

For cell cycle analysis PBL were collected, washed once with PBS and resuspended in 1 ml PBS. Cells were then fixed by drop-wise adding 9 ml fixing solution (1:1-mixture of acetone and 80% ethanol; stored at -20° C) while samples were gently mixed. Subsequently samples were kept on ice for 15 min and then at -20° C for at least 1h. PBL were then step-wise rehydrated and resuspended in 50 μg/ml propidium iodide solution with freshly added 50 μg/ml RNase A (Sigma-Aldrich). After incubation for 30 min at 37° C, the cells were analyzed by flow cytometry. Representative FACS plots are shown in Supplementary Figure 3.

### Western blot analysis

Cellular lysates were prepared and analyzed by Western Blotting as previously described [75]. Protein extracts were prepared by incubating the cells in lysis buffer (50 mM Tris, pH 7.4; 150 mM NaCl; 2 mM EGTA; 2 mM EDTA; 25 mM NaF; 25 mM β-glycerophosphate; 0.1 mM NaV; 0.2% Triton X-100; 0.3% Nonidet P40; proteinase inhibitor, Roche). Following centrifugation, protein concentrations of supernatants were determined by the BCA™ Protein Assay Kit (Thermo Scientific). 60 μg of protein per sample was separated electrophoretically using 8–15% SDS–PAGE gels and blotted onto Hybond™-C-Extra Nitrocellulose (GE Healthcare) or Immobilon-P Membrane (PVDF) (Merck Millipore) membranes. Proteins of interest were detected using the following antibodies: anti-α-Tubulin (mouse, monoclonal, DM1A, Abcam), anit-ATM (mouse, monoclonal, 5C2, Abcam), anti-BLM (rabbit, polyclonal, ab476, Abcam), anti-BRCA1 (mouse, monoclonal, MS110, Calbiochem), anti-CtIP (goat, polyclonal, T-16, Santa Cruz), anti-EXO1 (rabbit, polyclonal antibody GTX109891, GeneTex), anti-FEN1 (mouse, monoclonal, 21, BD BioSience), anti-GAPDH (mouse, monoclonal, ab9484, Abcam), anti-KU70 (mouse, monoclonal S5C11, Abcam), anti-LIG3 (rabbit, polyclonal, A1887, Abclonal), anti-LIG4 (rabbit, polyclonal, A1743, Abclonal), anti-MRE11 (rabbit, polyclonal, M-2, Novus), anti-p53 (mouse, monoclonal, DO-1, BD BioScience), anti-Rad51 (rabbit, polyclonal, H-92, Santa Cruz), anti-SIRT1 (rabbit, polyclonal, ab7343-100, Santa Cruz), anti-SIRT6 (rabbit, polyclonal, ab88494, Abcam), anti-WRN (rabbit, polyclonal, H-300, Santa Cruz), anti-XRCC4 (rabbit, polyclonal, A7539, Abclonal). Chemiluminescence signals were visualized using Clarity™ Western ECL Substrate (BioRad) and detected on a ChemiDocMP System (BioRad). Band intensities were quantified using ImageLab or ImageJ software. Intensity values of the protein of interest were corrected with the values of the corresponding loading control.

### Quantitative PCR

Total RNA was extracted from up to 10^6^ primary cultured PBL using the RNeasy plus mini kit (Qiagen) following the manufacturer’s instructions. Reverse transcription was performed applying the QuantiTect Reverse Transcription Kit (Qiagen). BLM, ATM and KU70 expression was measured by RT-qPCR using SensiFast Probes Lo-Rox Kit von Bioline (#BIO 84020) for BLM (#qHsaCEP0058401, Biorad), ATM (#qHsaCEP0052709, Biorad), KU70 (#qHsaCEP0055342, Biorad), PPIA (#qHsaCEP0041342, Biorad), HPRT1 (#Hs02800695_m1, Thermo), beta-2 (#4325797, Thermo), YWHAZ (#qHsaCIP0029093, Biorad), RPS17 (#qHsaCEP0041840, Biorad) and TBP (#qHsaCIP0036255, Biorad). PPIA, HPRT1, beta-2, YWHAZ, RPS17 and TBP served as housekeeping genes for normalization. Amplification and fluorescence signal detection was performed using the ViiA7 Real-Time PCR-System (Applied Biosystems, life technologies). Relative expression levels were calculated by the 2-ΔΔCT method [76].

### Statistics

Statistical analyses were performed using GraphPadPrism5.04 or GraphPadPrism8.0 software. PBL samples derived from different individuals were considered to be independent. To avoid mixing of independent (individual donors) and dependent (replicates within donors) values, mean values per individual were used for statistical analysis. The Mann-Whitney two-tailed test was applied for pairwise comparisons between PBL groups, defined by age and sex of the donors. For pairwise comparisons such as of controls versus irradiated PBL samples or knockdown versus control, Wilcoxon signed-rank test for matched pairs was used. Significance levels were not adjusted for multiple comparisons. Differences were considered as significant for p values < 0.05. For more detailed information on statistical results not preserved in the main text and including sample numbers for each experiment, see Supplementary Table 1. If not stated otherwise, significances calculated by Mann-Whitney two-tailed test are indicated by asterisks in the figures. n.s., p>0.1; trend, p<0.1; *, p<0.05; **, p<0.01; ***, p<0.001.

## Supplementary Material

Supplementary Figures

Supplementary Table 1

## References

[1] Ostan R, Monti D, Gueresi P, Bussolotto M, Franceschi C, Baggio G. Gender, aging and longevity in humans: an update of an intriguing/neglected scenario paving the way to a gender-specific medicine. Clin Sci (Lond). 2016; 130:1711–25. 10.1042/CS20160004 27555614 PMC4994139

[2] López-Otín C, Blasco MA, Partridge L, Serrano M, Kroemer G. The hallmarks of aging. Cell. 2013; 153:1194–217. 10.1016/j.cell.2013.05.039 23746838 PMC3836174

[3] Moskalev AA, Shaposhnikov MV, Plyusnina EN, Zhavoronkov A, Budovsky A, Yanai H, Fraifeld VE. The role of DNA damage and repair in aging through the prism of Koch-like criteria. Ageing Res Rev. 2013; 12:661–84. 10.1016/j.arr.2012.02.001 22353384

[4] Rübe CE, Fricke A, Widmann TA, Fürst T, Madry H, Pfreundschuh M, Rübe C. Accumulation of DNA damage in hematopoietic stem and progenitor cells during human aging. PLoS One. 2011; 6:e17487. 10.1371/journal.pone.0017487 21408175 PMC3049780

[5] Walter D, Lier A, Geiselhart A, Thalheimer FB, Huntscha S, Sobotta MC, Moehrle B, Brocks D, Bayindir I, Kaschutnig P, Muedder K, Klein C, Jauch A, et al. Exit from dormancy provokes DNA-damage-induced attrition in haematopoietic stem cells. Nature. 2015; 520:549–52. 10.1038/nature14131 25707806

[6] Ciccia A, Elledge SJ. The DNA damage response: making it safe to play with knives. Mol Cell. 2010; 40:179–204. 10.1016/j.molcel.2010.09.019 20965415 PMC2988877

[7] Petermann E, Helleday T. Pathways of mammalian replication fork restart. Nat Rev Mol Cell Biol. 2010; 11:683–87. 10.1038/nrm2974 20842177

[8] Himmels SF, Sartori AA. Controlling DNA-End Resection: An Emerging Task for Ubiquitin and SUMO. Front Genet. 2016; 7:152. 10.3389/fgene.2016.00152 27602047 PMC4993767

[9] Truong LN, Li Y, Shi LZ, Hwang PY, He J, Wang H, Razavian N, Berns MW, Wu X. Microhomology-mediated End Joining and Homologous Recombination share the initial end resection step to repair DNA double-strand breaks in mammalian cells. Proc Natl Acad Sci USA. 2013; 110:7720–25. 10.1073/pnas.1213431110 23610439 PMC3651503

[10] Mayer PJ, Lange CS, Bradley MO, Nichols WW. Gender differences in age-related decline in DNA double-strand break damage and repair in lymphocytes. Ann Hum Biol. 1991; 18:405–15. 10.1080/03014469100001702 1952798

[11] Garm C, Moreno-Villanueva M, Bürkle A, Petersen I, Bohr VA, Christensen K, Stevnsner T. Age and gender effects on DNA strand break repair in peripheral blood mononuclear cells. Aging Cell. 2013; 12:58–66. 10.1111/acel.12019 23088435 PMC4586247

[12] Patel J, Baptiste BA, Kim E, Hussain M, Croteau DL, Bohr VA. DNA damage and mitochondria in cancer and aging. Carcinogenesis. 2020; 41:1625–34. 10.1093/carcin/bgaa114 33146705 PMC7791626

[13] Li Z, Zhang W, Chen Y, Guo W, Zhang J, Tang H, Xu Z, Zhang H, Tao Y, Wang F, Jiang Y, Sun FL, Mao Z. Impaired DNA double-strand break repair contributes to the age-associated rise of genomic instability in humans. Cell Death Differ. 2016; 23:1765–77. 10.1038/cdd.2016.65 27391797 PMC5071568

[14] Lacoste S, Bhatia S, Chen Y, Bhatia R, O’Connor TR. Autologous hematopoietic stem cell transplantation in lymphoma patients is associated with a decrease in the double strand break repair capacity of peripheral blood lymphocytes. PLoS One. 2017; 12:e0171473. 10.1371/journal.pone.0171473 28207808 PMC5313139

[15] Anglada T, Genescà A, Martín M. Age-associated deficient recruitment of 53BP1 in G1 cells directs DNA double-strand break repair to BRCA1/CtIP-mediated DNA-end resection. Aging (Albany NY). 2020; 12:24872–93. 10.18632/aging.202419 33361520 PMC7803562

[16] Flach J, Bakker ST, Mohrin M, Conroy PC, Pietras EM, Reynaud D, Alvarez S, Diolaiti ME, Ugarte F, Forsberg EC, Le Beau MM, Stohr BA, Méndez J, et al. Replication stress is a potent driver of functional decline in ageing haematopoietic stem cells. Nature. 2014; 512:198–202. 10.1038/nature13619 25079315 PMC4456040

[17] Kraft D, Rall M, Volcic M, Metzler E, Groo A, Stahl A, Bauer L, Nasonova E, Salles D, Taucher-Scholz G, Bönig H, Fournier C, Wiesmüller L. NF-κB-dependent DNA damage-signaling differentially regulates DNA double-strand break repair mechanisms in immature and mature human hematopoietic cells. Leukemia. 2015; 29:1543–54. 10.1038/leu.2015.28 25652738

[18] Akyüz N, Boehden GS, Süsse S, Rimek A, Preuss U, Scheidtmann KH, Wiesmüller L. DNA substrate dependence of p53-mediated regulation of double-strand break repair. Mol Cell Biol. 2002; 22:6306–17. 10.1128/MCB.22.17.6306-6317.2002 12167722 PMC134001

[19] Bennardo N, Cheng A, Huang N, Stark JM. Alternative-NHEJ is a mechanistically distinct pathway of mammalian chromosome break repair. PLoS Genet. 2008; 4:e1000110. 10.1371/journal.pgen.1000110 18584027 PMC2430616

[20] Keimling M, Volcic M, Csernok A, Wieland B, Dörk T, Wiesmüller L. Functional characterization connects individual patient mutations in ataxia telangiectasia mutated (ATM) with dysfunction of specific DNA double-strand break-repair signaling pathways. FASEB J. 2011; 25:3849–60. 10.1096/fj.11-185546 21778326

[21] Keimling M, Deniz M, Varga D, Stahl A, Schrezenmeier H, Kreienberg R, Hoffmann I, König J, Wiesmüller L. The power of DNA double-strand break (DSB) repair testing to predict breast cancer susceptibility. FASEB J. 2012; 26:2094–104. 10.1096/fj.11-200790 22278937

[22] Deniz M, Romashova T, Kostezka S, Faul A, Gundelach T, Moreno-Villanueva M, Janni W, Friedl TW, Wiesmüller L. Increased single-strand annealing rather than non-homologous end-joining predicts hereditary ovarian carcinoma. Oncotarget. 2017; 8:98660–76. 10.18632/oncotarget.21720 29228718 PMC5716758

[23] Obermeier K, Sachsenweger J, Friedl TW, Pospiech H, Winqvist R, Wiesmüller L. Heterozygous PALB2 c.1592delT mutation channels DNA double-strand break repair into error-prone pathways in breast cancer patients. Oncogene. 2016; 35:3796–806. 10.1038/onc.2015.448 26640152 PMC4962030

[24] Mjelle R, Hegre SA, Aas PA, Slupphaug G, Drabløs F, Saetrom P, Krokan HE. Cell cycle regulation of human DNA repair and chromatin remodeling genes. DNA Repair (Amst). 2015; 30:53–67. 10.1016/j.dnarep.2015.03.007 25881042

[25] Wang C, Lees-Miller SP. Detection and repair of ionizing radiation-induced DNA double strand breaks: new developments in nonhomologous end joining. Int J Radiat Oncol Biol Phys. 2013; 86:440–49. 10.1016/j.ijrobp.2013.01.011 23433795 PMC3731128

[26] Pellegrino S, Michelena J, Teloni F, Imhof R, Altmeyer M. Replication-Coupled Dilution of H4K20me2 Guides 53BP1 to Pre-replicative Chromatin. Cell Rep. 2017; 19:1819–31. 10.1016/j.celrep.2017.05.016 28564601 PMC5857200

[27] Chatterjee N, Walker GC. Mechanisms of DNA damage, repair, and mutagenesis. Environ Mol Mutagen. 2017; 58:235–63. 10.1002/em.22087 28485537 PMC5474181

[28] Trenner A, Sartori AA. Harnessing DNA Double-Strand Break Repair for Cancer Treatment. Front Oncol. 2019; 9:1388. 10.3389/fonc.2019.01388 31921645 PMC6921965

[29] Tripathi V, Agarwal H, Priya S, Batra H, Modi P, Pandey M, Saha D, Raghavan SC, Sengupta S. MRN complex-dependent recruitment of ubiquitylated BLM helicase to DSBs negatively regulates DNA repair pathways. Nat Commun. 2018; 9:1016. 10.1038/s41467-018-03393-8 29523790 PMC5844875

[30] Croteau DL, Popuri V, Opresko PL, Bohr VA. Human RecQ helicases in DNA repair, recombination, and replication. Annu Rev Biochem. 2014; 83:519–52. 10.1146/annurev-biochem-060713-035428 24606147 PMC4586249

[31] Volcic M, Sparrer KM, Koepke L, Hotter D, Sauter D, Stürzel CM, Scherer M, Stamminger T, Hofmann TG, Arhel NJ, Wiesmüller L, Kirchhoff F. Vpu modulates DNA repair to suppress innate sensing and hyper-integration of HIV-1. Nat Microbiol. 2020; 5:1247–61. 10.1038/s41564-020-0753-6 32690953 PMC7616938

[32] Davalos AR, Kaminker P, Hansen RK, Campisi J. ATR and ATM-dependent movement of BLM helicase during replication stress ensures optimal ATM activation and 53BP1 focus formation. Cell Cycle. 2004; 3:1579–86. 10.4161/cc.3.12.1286 15539948

[33] Fischer KE, Riddle NC. Sex Differences in Aging: Genomic Instability. J Gerontol A Biol Sci Med Sci. 2018; 73:166–74. 10.1093/gerona/glx105 28575157 PMC5861920

[34] Li CH, Prokopec SD, Sun RX, Yousif F, Schmitz N, Boutros PC, and PCAWG Tumour Subtypes and Clinical Translation, and PCAWG Consortium. Sex differences in oncogenic mutational processes. Nat Commun. 2020; 11:4330. 10.1038/s41467-020-17359-2 32859912 PMC7455744

[35] Ju YJ, Lee KH, Park JE, Yi YS, Yun MY, Ham YH, Kim TJ, Choi HM, Han GJ, Lee JH, Lee J, Han JS, Lee KM, Park GH. Decreased expression of DNA repair proteins Ku70 and Mre11 is associated with aging and may contribute to the cellular senescence. Exp Mol Med. 2006; 38:686–93. 10.1038/emm.2006.81 17202845

[36] Espejel S, Klatt P, Ménissier-de Murcia J, Martín-Caballero J, Flores JM, Taccioli G, de Murcia G, Blasco MA. Impact of telomerase ablation on organismal viability, aging, and tumorigenesis in mice lacking the DNA repair proteins PARP-1, Ku86, or DNA-PKcs. J Cell Biol. 2004; 167:627–38. 10.1083/jcb.200407178 15545322 PMC2172587

[37] Li H, Vogel H, Holcomb VB, Gu Y, Hasty P. Deletion of Ku70, Ku80, or both causes early aging without substantially increased cancer. Mol Cell Biol. 2007; 27:8205–14. 10.1128/MCB.00785-07 17875923 PMC2169178

[38] Deniz M, Zengerling F, Gundelach T, Moreno-Villanueva M, Bürkle A, Janni W, Bolenz C, Kostezka S, Marienfeld R, Benckendorff J, Friedl TW, Wiesmüller L, Rall-Scharpf M. Age-related activity of Poly (ADP-Ribose) Polymerase (PARP) in men with localized prostate cancer. Mech Ageing Dev. 2021; 196:111494. 10.1016/j.mad.2021.111494 33887280

[39] Grube K, Bürkle A. Poly(ADP-ribose) polymerase activity in mononuclear leukocytes of 13 mammalian species correlates with species-specific life span. Proc Natl Acad Sci USA. 1992; 89:11759–63. 10.1073/pnas.89.24.11759 1465394 PMC50636

[40] Zaremba T, Thomas HD, Cole M, Coulthard SA, Plummer ER, Curtin NJ. Poly(ADP-ribose) polymerase-1 (PARP-1) pharmacogenetics, activity and expression analysis in cancer patients and healthy volunteers. Biochem J. 2011; 436:671–79. 10.1042/BJ20101723 21434873

[41] Grabarz A, Guirouilh-Barbat J, Barascu A, Pennarun G, Genet D, Rass E, Germann SM, Bertrand P, Hickson ID, Lopez BS. A role for BLM in double-strand break repair pathway choice: prevention of CtIP/Mre11-mediated alternative nonhomologous end-joining. Cell Rep. 2013; 5:21–28. 10.1016/j.celrep.2013.08.034 24095737

[42] Kijas AW, Lim YC, Bolderson E, Cerosaletti K, Gatei M, Jakob B, Tobias F, Taucher-Scholz G, Gueven N, Oakley G, Concannon P, Wolvetang E, Khanna KK, et al. ATM-dependent phosphorylation of MRE11 controls extent of resection during homology directed repair by signalling through Exonuclease 1. Nucleic Acids Res. 2015; 43:8352–67. 10.1093/nar/gkv754 26240375 PMC4787824

[43] Stork CT, Bocek M, Crossley MP, Sollier J, Sanz LA, Chédin F, Swigut T, Cimprich KA. Co-transcriptional R-loops are the main cause of estrogen-induced DNA damage. Elife. 2016; 5:e17548. 10.7554/eLife.17548 27552054 PMC5030092

[44] Schiewer MJ, Knudsen KE. Linking DNA Damage and Hormone Signaling Pathways in Cancer. Trends Endocrinol Metab. 2016; 27:216–25. 10.1016/j.tem.2016.02.004 26944914 PMC4808434

[45] Stender JD, Frasor J, Komm B, Chang KC, Kraus WL, Katzenellenbogen BS. Estrogen-regulated gene networks in human breast cancer cells: involvement of E2F1 in the regulation of cell proliferation. Mol Endocrinol. 2007; 21:2112–23. 10.1210/me.2006-0474 17550982

[46] Honour JW. Biochemistry of the menopause. Ann Clin Biochem. 2018; 55:18–33. 10.1177/0004563217739930 29027807

[47] Harman SM, Metter EJ, Tobin JD, Pearson J, Blackman MR, and Baltimore Longitudinal Study of Aging. Longitudinal effects of aging on serum total and free testosterone levels in healthy men. Baltimore Longitudinal Study of Aging. J Clin Endocrinol Metab. 2001; 86:724–31. 10.1210/jcem.86.2.7219 11158037

[48] Polkinghorn WR, Parker JS, Lee MX, Kass EM, Spratt DE, Iaquinta PJ, Arora VK, Yen WF, Cai L, Zheng D, Carver BS, Chen Y, Watson PA, et al. Androgen receptor signaling regulates DNA repair in prostate cancers. Cancer Discov. 2013; 3:1245–53. 10.1158/2159-8290.CD-13-0172 24027196 PMC3888815

[49] Martin-Hernandez K, Rodriguez-Vargas JM, Schreiber V, Dantzer F. Expanding functions of ADP-ribosylation in the maintenance of genome integrity. Semin Cell Dev Biol. 2017; 63:92–101. 10.1016/j.semcdb.2016.09.009 27670719

[50] Cunniff C, Bassetti JA, Ellis NA. Bloom’s Syndrome: Clinical Spectrum, Molecular Pathogenesis, and Cancer Predisposition. Mol Syndromol. 2017; 8:4–23. 10.1159/000452082 28232778 PMC5260600

[51] Chambers SM, Shaw CA, Gatza C, Fisk CJ, Donehower LA, Goodell MA. Aging hematopoietic stem cells decline in function and exhibit epigenetic dysregulation. PLoS Biol. 2007; 5:e201. 10.1371/journal.pbio.0050201 17676974 PMC1925137

[52] Symington LS. Mechanism and regulation of DNA end resection in eukaryotes. Crit Rev Biochem Mol Biol. 2016; 51:195–212. 10.3109/10409238.2016.1172552 27098756 PMC4957645

[53] Bugreev DV, Mazina OM, Mazin AV. Bloom syndrome helicase stimulates RAD51 DNA strand exchange activity through a novel mechanism. J Biol Chem. 2009; 284:26349–59. 10.1074/jbc.M109.029371 19632996 PMC2786030

[54] Karow JK, Constantinou A, Li JL, West SC, Hickson ID. The Bloom’s syndrome gene product promotes branch migration of holliday junctions. Proc Natl Acad Sci USA. 2000; 97:6504–08. 10.1073/pnas.100448097 10823897 PMC18638

[55] Patel DS, Misenko SM, Her J, Bunting SF. BLM helicase regulates DNA repair by counteracting RAD51 loading at DNA double-strand break sites. J Cell Biol. 2017; 216:3521–34. 10.1083/jcb.201703144 28912125 PMC5674892

[56] Bachrati CZ, Borts RH, Hickson ID. Mobile D-loops are a preferred substrate for the Bloom’s syndrome helicase. Nucleic Acids Res. 2006; 34:2269–79. 10.1093/nar/gkl258 16670433 PMC1456333

[57] Wang Y, Smith K, Waldman BC, Waldman AS. Depletion of the bloom syndrome helicase stimulates homology-dependent repair at double-strand breaks in human chromosomes. DNA Repair (Amst). 2011; 10:416–26. 10.1016/j.dnarep.2011.01.009 21300576 PMC3062690

[58] Gaymes TJ, North PS, Brady N, Hickson ID, Mufti GJ, Rassool FV. Increased error-prone non homologous DNA end-joining--a proposed mechanism of chromosomal instability in Bloom’s syndrome. Oncogene. 2002; 21:2525–33. 10.1038/sj.onc.1205331 11971187

[59] So S, Adachi N, Lieber MR, Koyama H. Genetic interactions between BLM and DNA ligase IV in human cells. J Biol Chem. 2004; 279:55433–42. 10.1074/jbc.M409827200 15509577

[60] Onclercq-Delic R, Calsou P, Delteil C, Salles B, Papadopoulo D, Amor-Guéret M. Possible anti-recombinogenic role of Bloom’s syndrome helicase in double-strand break processing. Nucleic Acids Res. 2003; 31:6272–82. 10.1093/nar/gkg834 14576316 PMC275476

[61] Langland G, Elliott J, Li Y, Creaney J, Dixon K, Groden J. The BLM helicase is necessary for normal DNA double-strand break repair. Cancer Res. 2002; 62:2766–70. 12019152

[62] Mendez-Dorantes C, Tsai LJ, Jahanshir E, Lopezcolorado FW, Stark JM. BLM has Contrary Effects on Repeat-Mediated Deletions, based on the Distance of DNA DSBs to a Repeat and Repeat Divergence. Cell Rep. 2020; 30:1342–57.e4. 10.1016/j.celrep.2020.01.001 32023454 PMC7085117

[63] Beamish H, Kedar P, Kaneko H, Chen P, Fukao T, Peng C, Beresten S, Gueven N, Purdie D, Lees-Miller S, Ellis N, Kondo N, Lavin MF. Functional link between BLM defective in Bloom’s syndrome and the ataxia-telangiectasia-mutated protein, ATM. J Biol Chem. 2002; 277:30515–23. 10.1074/jbc.M203801200 12034743

[64] Dorak MT, Karpuzoglu E. Gender differences in cancer susceptibility: an inadequately addressed issue. Front Genet. 2012; 3:268. 10.3389/fgene.2012.00268 23226157 PMC3508426

[65] Zhang Y, Rowley JD. Chromatin structural elements and chromosomal translocations in leukemia. DNA Repair (Amst). 2006; 5:1282–97. 10.1016/j.dnarep.2006.05.020 16893685

[66] Anglada T, Repullés J, Espinal A, LaBarge MA, Stampfer MR, Genescà A, Martín M. Delayed γH2AX foci disappearance in mammary epithelial cells from aged women reveals an age-associated DNA repair defect. Aging (Albany NY). 2019; 11:1510–23. 10.18632/aging.101849 30875333 PMC6428106

[67] Sedelnikova OA, Horikawa I, Redon C, Nakamura A, Zimonjic DB, Popescu NC, Bonner WM. Delayed kinetics of DNA double-strand break processing in normal and pathological aging. Aging Cell. 2008; 7:89–100. 10.1111/j.1474-9726.2007.00354.x 18005250

[68] Sharma PM, Ponnaiya B, Taveras M, Shuryak I, Turner H, Brenner DJ. High throughput measurement of γH2AX DSB repair kinetics in a healthy human population. PLoS One. 2015; 10:e0121083. 10.1371/journal.pone.0121083 25794041 PMC4368624

[69] Marková E, Schultz N, Belyaev IY. Kinetics and dose-response of residual 53BP1/gamma-H2AX foci: co-localization, relationship with DSB repair and clonogenic survival. Int J Radiat Biol. 2007; 83:319–29. 10.1080/09553000601170469 17457757

[70] Rothkamm K, Barnard S, Moquet J, Ellender M, Rana Z, Burdak-Rothkamm S. DNA damage foci: Meaning and significance. Environ Mol Mutagen. 2015; 56:491–504. 10.1002/em.21944 25773265

[71] Siddiqui MS, François M, Fenech MF, Leifert WR. Persistent γH2AX: A promising molecular marker of DNA damage and aging. Mutat Res Rev Mutat Res. 2015; 766:1–19. 10.1016/j.mrrev.2015.07.001 26596544

[72] Sedelnikova OA, Redon CE, Dickey JS, Nakamura AJ, Georgakilas AG, Bonner WM. Role of oxidatively induced DNA lesions in human pathogenesis. Mutat Res. 2010; 704:152–59. 10.1016/j.mrrev.2009.12.005 20060490 PMC3074954

[73] Michelena J, Pellegrino S, Spegg V, Altmeyer M. Replicated chromatin curtails 53BP1 recruitment in BRCA1-proficient and BRCA1-deficient cells. Life Sci Alliance. 2021; 4:e202101023. 10.26508/lsa.202101023 33811064 PMC8046418

[74] Kamentsky L, Jones TR, Fraser A, Bray MA, Logan DJ, Madden KL, Ljosa V, Rueden C, Eliceiri KW, Carpenter AE. Improved structure, function and compatibility for CellProfiler: modular high-throughput image analysis software. Bioinformatics. 2011; 27:1179–80. 10.1093/bioinformatics/btr095 21349861 PMC3072555

[75] Hampp S, Kiessling T, Buechle K, Mansilla SF, Thomale J, Rall M, Ahn J, Pospiech H, Gottifredi V, Wiesmüller L. DNA damage tolerance pathway involving DNA polymerase ι and the tumor suppressor p53 regulates DNA replication fork progression. Proc Natl Acad Sci USA. 2016; 113:E4311–19. 10.1073/pnas.1605828113 27407148 PMC4968756

[76] Livak KJ, Schmittgen TD. Analysis of relative gene expression data using real-time quantitative PCR and the 2(-Delta Delta C(T)) Method. Methods. 2001; 25:402–08. 10.1006/meth.2001.1262 11846609

