# Supplementary material for: Sex-specific differences in DNA double-strand break repair of cycling human lymphocytes during aging
Source: Aging (Albany NY). 2021 Sep 10;13(17):21066–89. doi: 10.18632/aging.203519 (PMC8457596; doi:10.18632/aging.203519)
Supplement: Supplementary Figures [file aging-13-203519-s001.pdf]

## SUPPLEMENTARY FIGURES

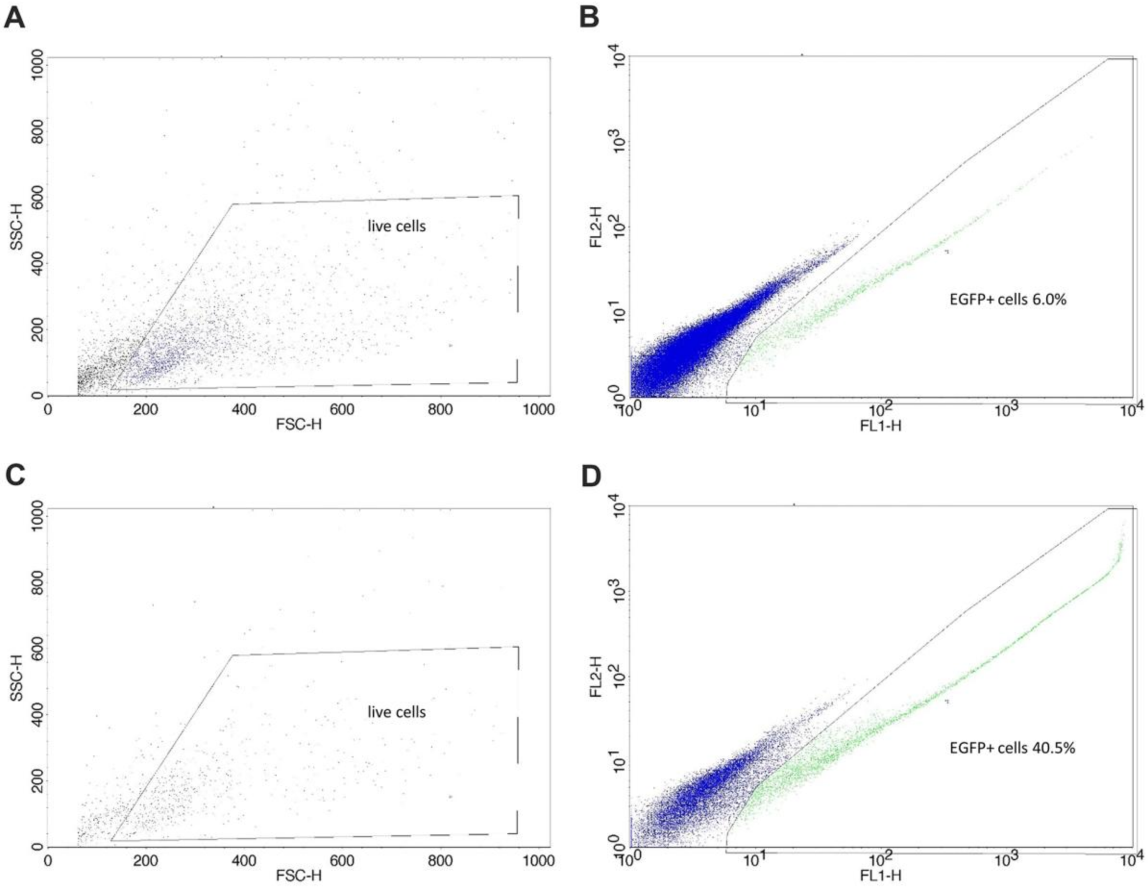


**E**

$$\text{DSB repair frequency [\%]} = \left( \frac{\text{repair events/live cells*100}}{\text{transfection efficiency (\%)}} \right) \text{*100}$$

**Supplementary Figure 1. EGFP based DSB repair assay.** (**A**–**C**) Exemplary FACS plots of PBL nucleofected with I-*SceI* meganuclease expression plasmid, pCMV-I-Sce-I and pBS (**A**, **B**) (determination of repair frequency) or wtEGFP expression plasmid (**C**, **D**) (determination of transfection efficiency) and NHEJ substrate (plasmid EJ5SceGFP). The frequencies of EGFP-positive cells were detected by flow cytometry 24h post transfection. (**A**, **C**) The live cell population was gated in a forward scatter (FSC-H)/ side scatter (SSC-H) dot plot. (**B**, **D**) The DSB repair frequency was determined as the fraction of green fluorescent cells within the whole live cell population using a diagonal gating method in the FLI-H/FL2-H dot plot and corrected for the transfection efficiency in the split sample. (**E**) Formula for DSB repair frequency calculation.

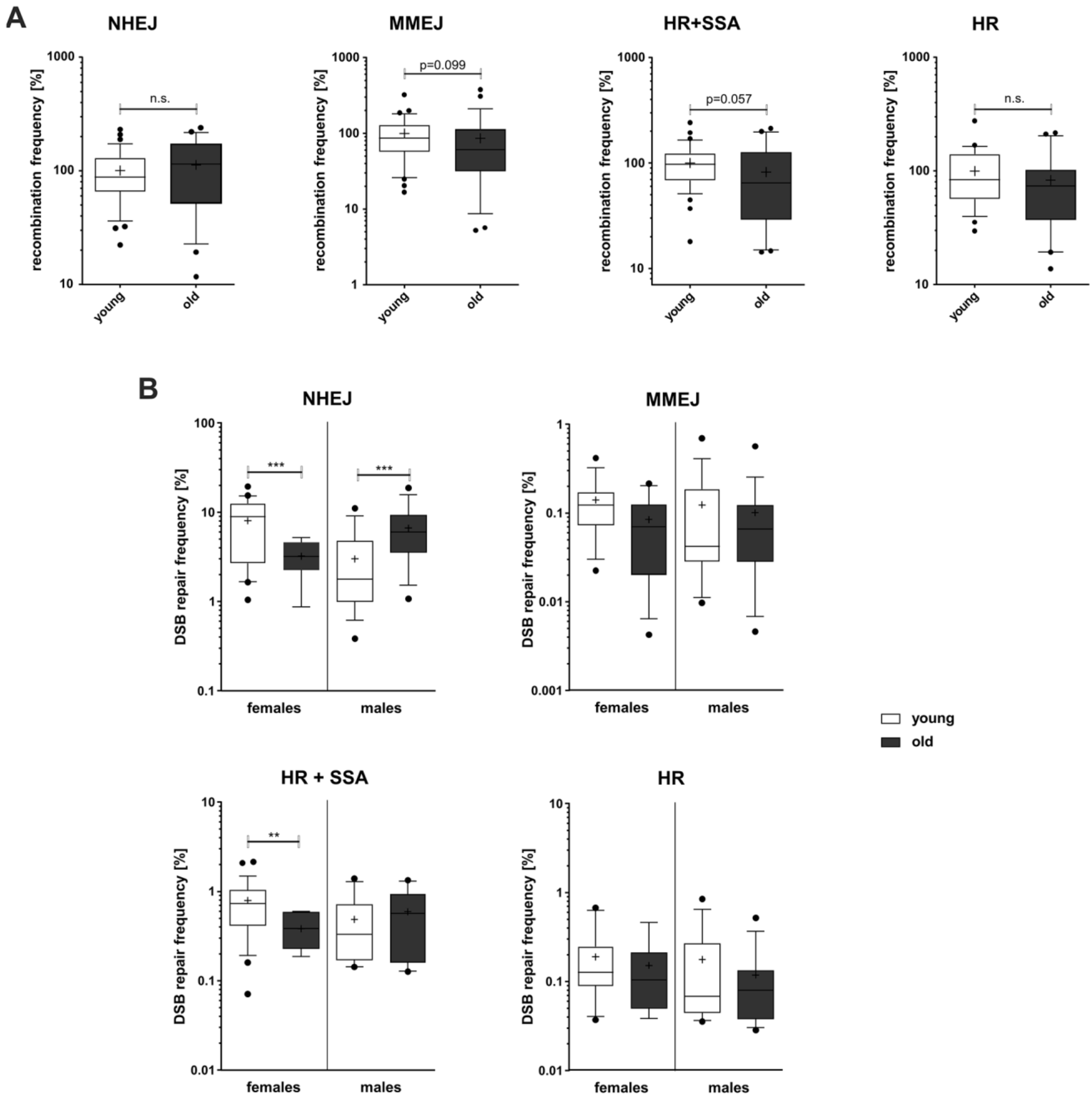


**Supplementary Figure 2. Additional analyses on DSB repair pathway activities in PBL from different age groups.** DSB repair activity measurements by NHEJ, MMEJ, HR+SSA and HR are shown in box plots with mean value (cross), median (line) and 95% CI (whiskers). (**A**) DSB repair frequencies in PBL from young and old donors of both sexes were normalized to the mean of young donors for each experimental day. n.s., p>0.1; Mann Whitney test. (**B**) Absolute DSB repair frequencies. **, p>0.01; ***. p>0.001; n.s., p>0.1; Mann Whitney test.

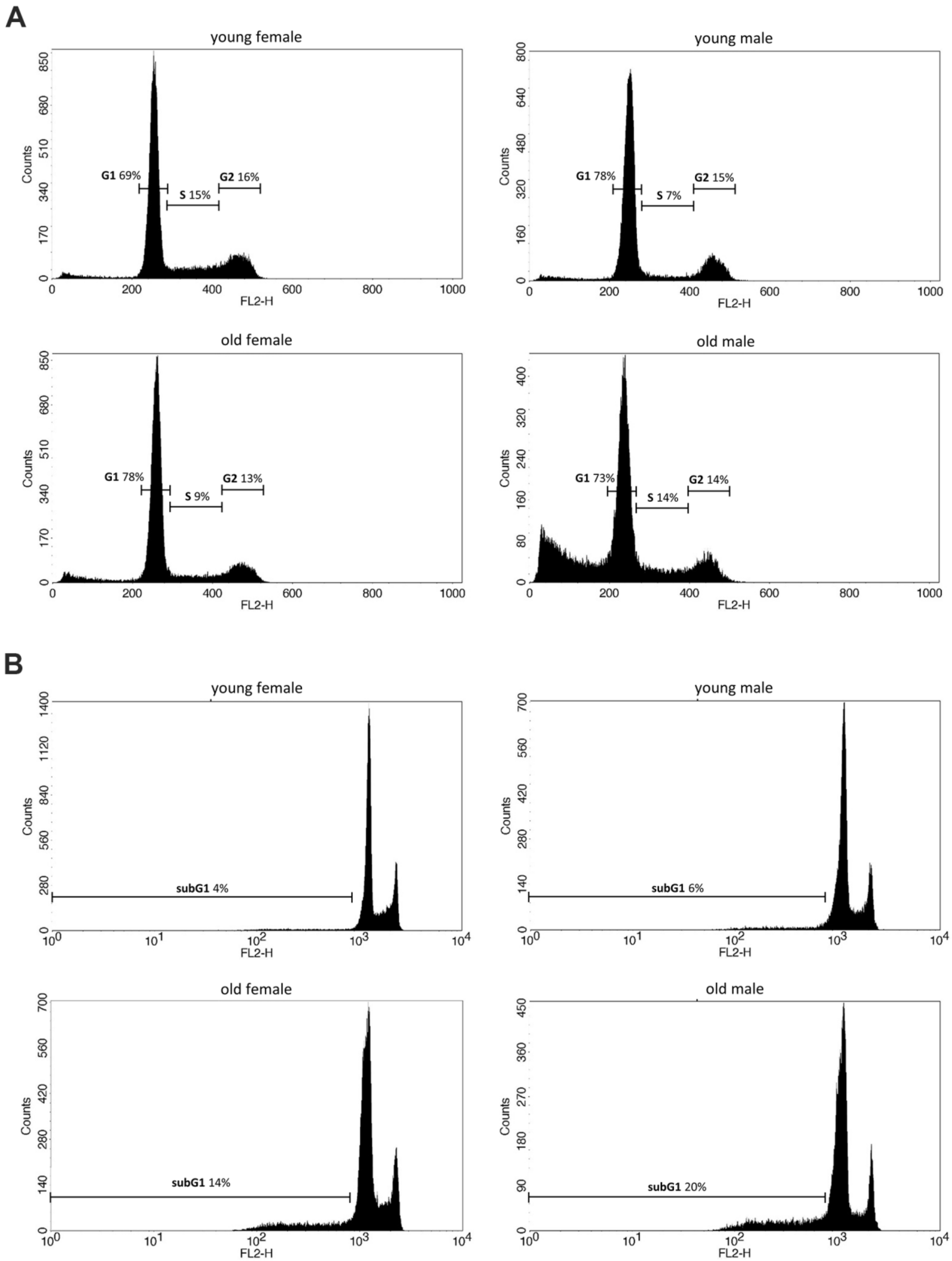


**Supplementary Figure 3. Cell cycle and cell death analysis.** Exemplary histograms of PBL cultured for 72h followed by PI staining and flow cytometry analysis. Cells were detected in FL2-H histogram using a linear scale for cell cycle distribution analysis (**A**) and using a logarithmic scale for evaluation of the subG1 fraction (**B**).

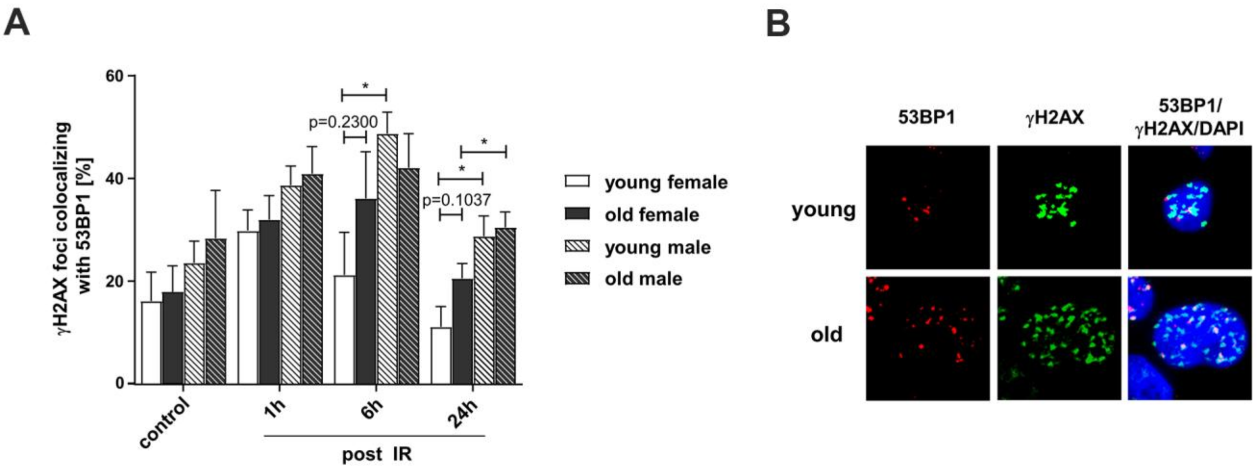


**Supplementary Figure 4. Colocalisation of γH2AX with 53BP1 foci.** Irradiated PBL were re-cultivated and fixed at indicated time points. γH2AX and 538P1 were immunocytochemically detected and the numbers of γH2AX foci colocalizing (col.) with 53BP1 foci were scored in 50-200 nuclei from 8 donors per group. (**A**) The mean percentages of γH2AX foci colocalizing with 53BP1 are show in columns with SEM. (**B**) Exemplary immunofluorescence images of nuclei with IR-induced 53BP1 and γH2AX foci 1h post IR.

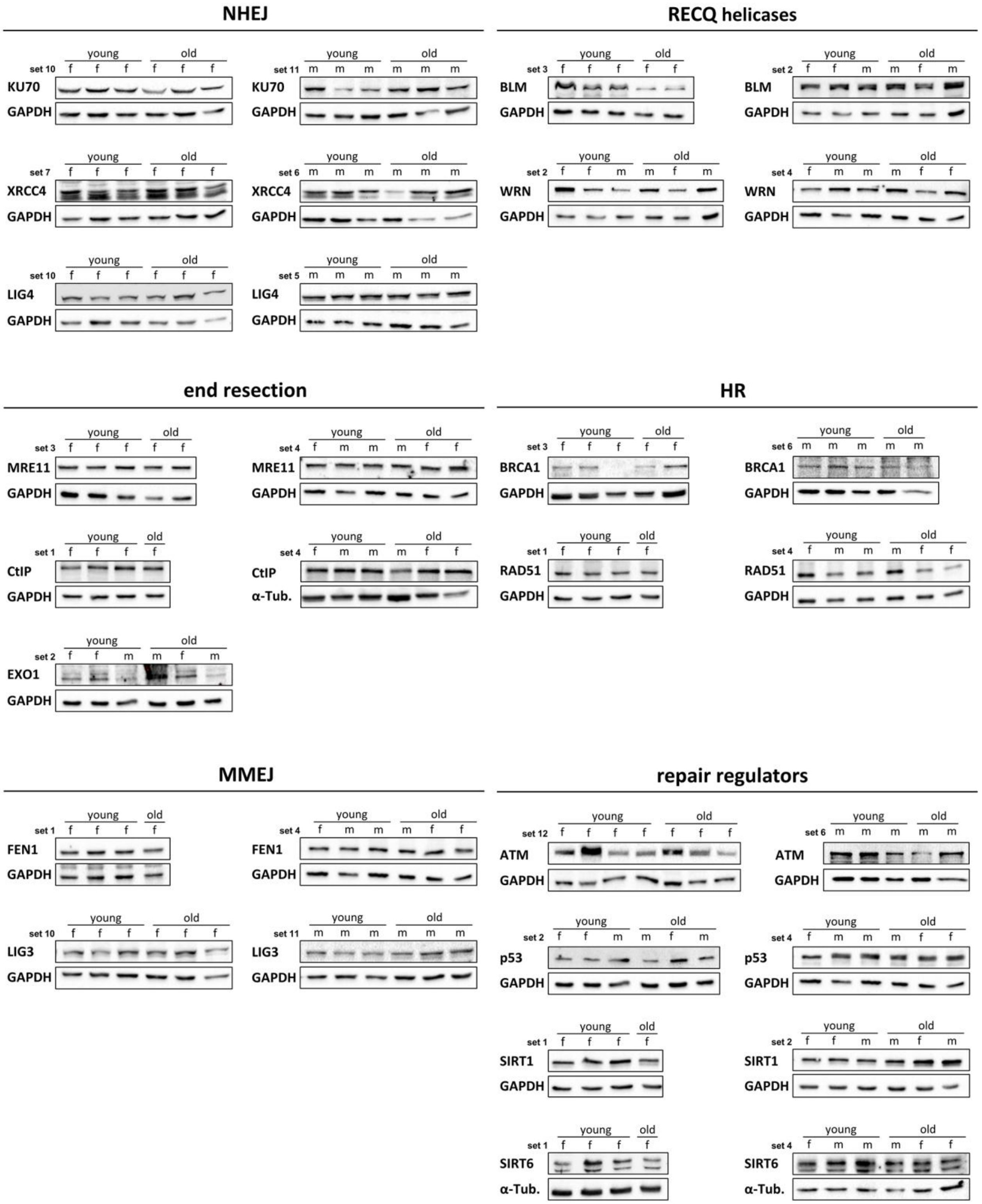


**Supplementary Figure 5. Detection of DDR factors by western blotting.** Protein lysates were prepared from primary PBL cultures and distributed to 12 sets, each containing samples from both age groups. Western blot analysis was performed to determine protein levels of NHEJ factors (KU70, LIG3, LIG4, XRCC4), end resection factors (MRE11, CtIP), MMEJ factors (FEN1, LIG3), HR factors (BRCA1, RAD51), RECQ helicases (BLM, WRN), repair regulators and aging associated factors (ATM, p53, SIRT1, SIRT6), GAPDH or α-Tubulin (α-Tub.) served as loading controls. Shown are representative Western Blots. To save scarce sample material several proteins were detected on the same membrane and thus the same loading controls are shown several times.

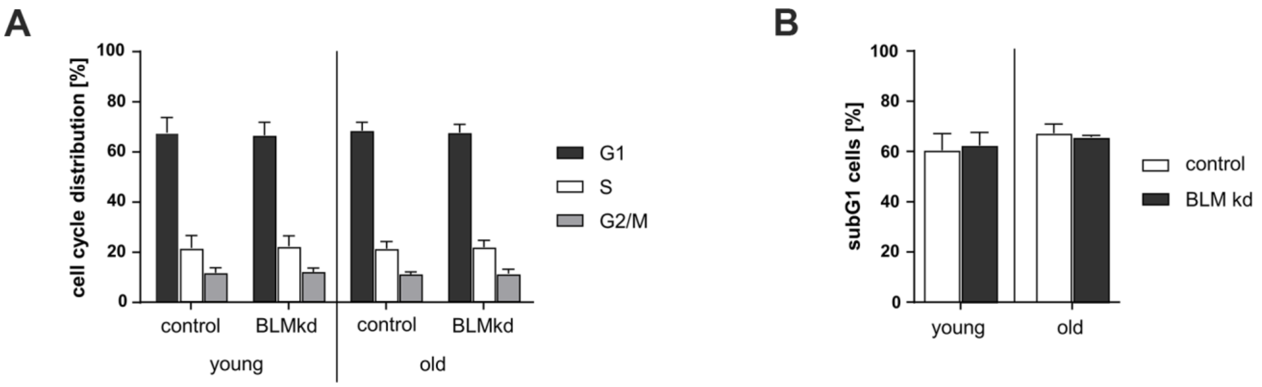


**Supplementary Figure 6. Cell cycle distribution and cell death in PBL after BLM knockdown.** Cultivated PBL were nucleofected with a DNA mixture containing pCMV-I-SceI, repair substrate EJ-EGFP (MMEJ), PBS or wild-type EGFP expression plasmid and knockdown (kd) plasmids silencing BLM or empty vector controls. 24h post nucleofection PBL were fixed and DNA content analyzed by propidium iodide staining and flow cytometry. Percentage of live cells in G1-, S-, and G2-phase (**A**) and proportion of dead cells, determined by subG1-DNA content (**B**). Columns, mean values; bars, SD; female: n=4 (young), n=4 (old); Wilcoxon matched-pairs signed rank test (Supplementary Table 1).
